# Pharmaceutical policy and off‐label prescribing in pregnancy: A population‐based historical cohort study analyzing inequality in access to antiemetics within Australia's Pharmaceutical Benefits Scheme

**DOI:** 10.1111/aogs.70283

**Published:** 2026-06-11

**Authors:** Hannah Jackson, Luke Grzeskowiak, Joanne Enticott, Sarah Wise, Emily Callander

**Affiliations:** ^1^ Faculty of Health, School of Public Health University of Technology Sydney Sydney New South Wales Australia; ^2^ College of Medicine and Public Health Flinders University Adelaide South Australia Australia; ^3^ SAHMRI Women and Kids, South Australian Health and Medical Research Institute Adelaide South Australia Australia; ^4^ Monash Centre for Health Research and Implementation (MCHRI), Faculty of Medicine, Nursing and Health Sciences Monash University Clayton Victoria Australia

**Keywords:** costs and cost analysis, dataset, equity, maternal health, medication, obstetric

## Abstract

**Introduction:**

Nausea and vomiting in pregnancy is highly prevalent and can significantly impact pregnant women's quality of life. Despite this, access to effective pharmacotherapies can be constrained by stringent regulatory controls and socioeconomic barriers. The objective of this study was to examine the socioeconomic distribution of antiemetics (metoclopramide, ondansetron, and prochlorperazine) dispensed to pregnant women through Australia's publicly subsidized Pharmaceutical Benefits Scheme.

**Material and Methods:**

We used the Maternity1000 linked administrative dataset to characterize antiemetics dispensed during 297 630 pregnancies in Queensland, Australia (July 2013 to June 2018). Using a population‐based historical cohort study design, we analyzed dispensing volume, prevalence, and government expenditure across socioeconomic quintiles, with socioeconomic disadvantage defined using the Australian Bureau of Statistics' Index of Relative Socioeconomic Disadvantage. Inequalities in medication access and public expenditure were assessed using concentration indices (C) and concentration curves.

**Results:**

Off‐label ondansetron dispensings for nausea and vomiting in pregnancy (i.e., use outside Therapeutic Goods Administration‐approved indications and not subsidized under the Pharmaceutical Benefits Scheme) accounted for the largest share of public expenditure (53.5%), followed by metoclopramide (45.2%) and prochlorperazine (1.3%). Across all three antiemetics, prevalence was highest among women in the most socioeconomically disadvantaged quintiles and declined progressively across the two least disadvantaged groups. Small pro‐poor inequalities in access (C < −0.10) and moderate pro‐poor inequalities in public expenditure (C > −0.25) were observed across all antiemetics. (Medication access: C_metoclopramide_ = −0.07, 95% CI (−0.080 to −0.068); C_ondansetron_ = −0.09, 95% CI (−0.114 to −0.075); C_prochlorperazine_ = −0.08, 95% CI (−0.109 to −0.045). Government expenditure: C_metoclopramide_ = −0.30, 95% CI (−0.316 to −0.285); C_ondansetron_ = −0.25, 95% CI (−0.297 to −0.198); C_prochlorperazine_ = −0.28, 95% CI (−0.350 to −0.205)).

**Conclusions:**

Off‐label ondansetron access accounted for the majority of public expenditure on antiemetics dispensed during pregnancy, revealing a disconnect between health policy, clinical practice, public expenditure, and pregnant women's needs. While pro‐poor access and public subsidies for antiemetics align with the equity elements embedded in the design of the Pharmaceutical Benefits Scheme, they may also be reflective of inequitable access to other unsubsidized, guideline‐recommended pharmacotherapies for nausea and vomiting in pregnancy.

## Graphical abstract

Public spending on antiemetics among pregnant women in Australia is concentrated in socioeconomically disadvantaged women. However, public expenditure is also driven by off‐label ondansetron use (i.e., use outside regulatory‐approved indications), highlighting a misalignment between pharmaceutical policy, public expenditure patterns, and pregnant women's therapeutic needs.

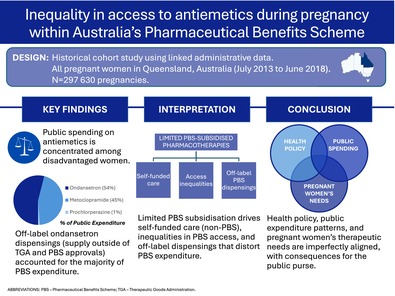

**Abbreviations**
95% CI: 95% confidence interval
C: concentration index
HG: hyperemesis gravidarum
IRSD: Index of Relative Socioeconomic Disadvantage
NVP: nausea and vomiting in pregnancy
PBS: Pharmaceutical Benefits Scheme
QPDC: Queensland Perinatal Data Collection

**Key message**

Socioeconomic barriers can limit access to antiemetics during pregnancy. This study demonstrates greater access and distribution of public funding among disadvantaged women. Off‐label ondansetron access dominated public expenditure, revealing misalignment between pharmaceutical policy, public expenditure patterns, and pregnant women's needs.

## 1 INTRODUCTION

Nausea and vomiting in pregnancy (NVP) affects between 50% and 80% of pregnant women.^1^, ^2^ A more severe form, hyperemesis gravidarum (HG), affects between 0.3% and 3.6% of pregnancies and involves severe, intractable nausea and vomiting often requiring hospital admission and intensive pharmacotherapeutic management.^3^ Both NVP and HG can severely reduce maternal well‐being, limiting a woman's capacity to work, care for others, manage domestic tasks, and participate socially.^4^ They also impose a substantial economic burden on health systems, families, and society through lost productivity, reliance on external carers, increased health care costs, and, in severe cases, recurring hospitalization.^5^ Timely access to appropriate support and effective treatments for NVP and HG should be a key priority for healthcare providers, policymakers, and the pharmaceutical industry to minimize avoidable suffering and mitigate associated health and economic consequences.

Despite its high prevalence, limited progress has been made in developing therapeutic options for NVP that are equitably accessible to pregnant women.^6^ Evidence suggests that only 10% to 18% of pregnant women experiencing NVP use conventional medications to manage their symptoms.^7^ A major contributor to this treatment gap is the enduring impact of the thalidomide tragedy of the 1960s, which resulted in severe birth defects among thousands of infants exposed in utero.^8^ In response, stringent regulatory frameworks and toxicity testing requirements were introduced to protect pregnant women and their babies. While these safeguards have been critical in preventing further medication‐induced harms, they have also contributed to a persistent knowledge gap regarding the safety of medications used during pregnancy and have discouraged the development of new medications, and regulatory approval of existing therapies, for common antenatal conditions.^9^

This historical and regulatory context has contributed to ongoing reluctance among pharmaceutical companies to seek regulatory approval for pregnancy indications, resulting in many antiemetic therapies being used off‐label during pregnancy (i.e., prescribing a medication outside of the regulatory‐approved therapeutic indication) or being excluded from public subsidy schemes.^10^, ^11^ Economic forces that shape the development, regulation, and subsidization of antiemetic medicines frequently neglect to cater for the clinical realities experienced by pregnant women and prescribers, resulting in a plethora of regulatory, financial, and service‐level access barriers.^12^ Although equitable access to essential medications during pregnancy has received increasing attention,^13^, ^14^, ^15^ progress remains constrained by the persistent norm of shielding pregnant women from research rather than generating evidence to support safe, effective, and accessible prescribing. This contributes to widespread reliance on off‐label prescribing (eg ondansetron for NVP and HG)^16^, ^17^ exposing medical practitioners, pregnant women, and unborn babies to unknown risks in the absence of clinical trials data and regulatory safeguards.^18^ Additionally, when medications fall outside of approved reimbursement pathways, financial barriers to access for socioeconomically disadvantaged women are heightened. An Australian study previously showed some pregnant women access ondansetron through the country's universal medicines subsidy scheme, the Pharmaceutical Benefits Scheme (PBS), despite its lack of approval for NVP via this pathway.^19^ This phenomenon, known as “PBS leakage”, raises important questions about how regulatory pathways and socioeconomic status shape access to pregnancy‐relevant medications.

This study aimed to examine pregnant women's access to antiemetic medications in Australia, where pharmaceuticals are publicly subsidized through a well‐established pharmaceutical access program—the PBS. Specifically, it investigates socioeconomic disparities in both the distribution of access to antiemetics and the associated public expenditure under PBS funding provisions.

## 2 MATERIAL AND METHODS

### 2.1 Study design, population, and setting

This population‐level historical cohort study utilized an existing linked administrative dataset, Maternity1000,^20^ to analyze medication access data for every pregnant woman who gave birth in Queensland, Australia, between 1st July 2013 and 30th June 2018. Queensland Perinatal Data Collection (QPDC) records were linked with PBS claims and cost records from 1st September 2012 to 30th June 2018. Women are included in the QPDC dataset if they gave birth to live babies, or stillborn babies of ≥ 20 weeks gestation or ≥ 400 g.^21^ Women appear multiple times if they had more than one pregnancy that met QPDC recording criteria during the study timeframe. Births occurring in public and private organizations are included. The study relied on deidentified, population‐level linked administrative health care data, making individual informed consent impractical. Data access and use were approved under the Public Health Act, with ethics approval obtained from the Townsville Hospital and Health Service Human Research Ethics Committee, the Australian Institute of Health and Welfare, and the University of Technology Sydney. Births with an unknown birth year were excluded from the analysis (*n* = 93). All prescriptions dispensed under the funding provisions of the PBS have been included in this analysis (including under co‐payment dispensings and prescriptions dispensed under the Closing The Gap PBS Co‐payment Program). Data was available for 237 540 women (297 630 pregnancies).

### 2.2 Australia's Pharmaceutical Benefits Scheme (PBS)

The PBS is a key mechanism for achieving the goals of Australia's National Medicines Policy—particularly equitable, timely, safe, and affordable access to necessary medicines.^22^ As the country's primary program for subsidized medication access, the PBS reflects the National Medicines Policy's focus on equitable access and improved health outcomes, especially for vulnerable population groups including those of low socioeconomic status and pregnant women. To be listed on the PBS, medications must be approved by the Therapeutic Goods Administration and recommended for subsidy by the Pharmaceutical Benefits Advisory Committee. As at 1st January 2025, general patients pay up to $31.60 per medicine, and concessional patients $7.70; with the remainder covered by the Australian Government.^23^ In June 2024, 930 different medications were approved for PBS subsidy.^24^

### 2.3 Antiemetic medications for NVP

Table 1 summarizes recommended medications for NVP according to the Society of Obstetric Medicine of Australia and New Zealand and the Australian Therapeutic Guidelines, along with their PBS availability.^25^, ^26^ Notably, no first‐line medications are subsidized under the PBS; they are only available over‐the‐counter. This study examines any inequality in pregnant women's access to PBS‐subsidized antiemetic medications. Therefore, only dispensings of metoclopramide, ondansetron, and prochlorperazine have been included. Promethazine was excluded since the oral formulation is not PBS‐listed, and the number of parenteral dispensings was too low (<10) to uphold privacy protection criteria. Additionally, prednisolone was excluded because the therapeutic indication for its use could not reliably be attributed to NVP.

**TABLE 1:** Recommended antiemetics for nausea and vomiting in pregnancy (NVP) and their availability via the Pharmaceutical Benefits Scheme (PBS).

Antiemetic	Dose and route	PBS availability
***1.1 First‐line pharmacological management***		
Ginger	Use standardized products (as opposed to foods) orally up to 1200 mg/day (in divided doses)	Not available via the PBS
+/−		
Pyridoxine^a^	10–50 mg orally three to four times a day (maximum 200 mg/day)	Not available via the PBS
+/−		
Doxylamine^a^	25 mg orally at night (which may be further increased as tolerated)	Not available via the PBS
***1.2 If NVP symptoms persist***		
*Additional antiemetics may be introduced in a stepwise manner depending on severity of symptoms, medication tolerability, and effectiveness*.		
Metoclopramide	10 mg (oral/ parenteral) every 8 h as required	PBS‐listed (General Schedule; unrestricted benefit)^b^
Ondansetron	4–8 mg (oral/ parenteral) every 8 to 12 h as required	PBS‐listed (General Schedule; **restricted** benefit)
		Approved only for chemotherapy/ radiotherapy‐induced nausea and vomiting. Not PBS‐approved for NVP
Prochlorperazine	5–10 mg orally every 6 to 8 h or 12.5 mg parenterally every 8 h as required^c^	PBS‐listed (General Schedule; unrestricted benefit)
Promethazine	10–25 mg orally every 6 to 8 h as required	Not available via the PBS^d^

^a^ Combination doxylamine PLUS pyridoxine products are accessible internationally but are not available in Australia.

^b^ ‘Unrestricted benefit’: no restrictions imposed on therapeutic use.

^c^ Prochlorperazine suppositories were available on the PBS during the study time frame and have been included in the analysis.

^d^ Injectable promethazine is PBS‐listed but is not typically used for NVP in community‐based settings.

### 2.4 Socioeconomic groupings

Socioeconomic status for each woman was determined by mapping the postcode recorded in the Queensland Perinatal Data Collection as the mother's usual place of residence to the Australian Bureau of Statistics' Index of Relative Socioeconomic Disadvantage (IRSD) 2016.^27^ The IRSD ranks areas on a continuous scale according to multiple measures of socioeconomic disadvantage, including low annual household equivalised income (<$26 000), jobless families (unemployed parents living with children under 15 years), lack of internet access, and single‐parent households.^27^ Low IRSD scores indicate a large proportion of disadvantaged residents, and high scores indicate a relatively low proportion of disadvantaged residents. IRSD scores have been further categorized into deciles and quintiles to aid presentation and interpretation of results. Pregnancies were excluded if an IRSD could not be assigned (*n* = 179 for error in postcode recorded; *n* = 51 for postcode without an IRSD assigned).

### 2.5 Statistical analysis

All statistical analyses were performed using SAS Version 9.4. Data analysis was conducted within the secure online platform Secure Unified Research Environment (SURE), operated by the Sax Institute.^28^ The unit of analysis for this study was per pregnancy; therefore, cluster‐robust standard errors were used in the concentration index analyses to account for women with more than one pregnancy. There are difficulties associated with assigning the initial date of a pregnancy within our dataset, as the end date for pregnancy (i.e., the date of delivery) is recorded as the month and year of birth, with the date being recorded as the 1st of the month in each instance (as a privacy protection mechanism). Consequently, dispensings in the final month of pregnancy may be missed. The start date for each pregnancy was calculated by subtracting the gestation period (in weeks and days) from the assigned date of delivery. To minimize misclassification of dispensings that did not occur during early pregnancy, we chose to exclude any events occurring in the first 30 days of pregnancy.

Descriptive statistics summarize demographic characteristics and are shown as counts and percentages; stratified by socioeconomic quintile. Missing data was explicitly reported as missing in the results. Descriptive statistics were also used to summarize the distribution of access to antiemetic medications for NVP across each socioeconomic quintile. Quantity, prevalence, and Government expenditure on antiemetic medications dispensed to pregnant women according to socioeconomic quintile are reported. Mean Government cost per dispensing with 95% confidence intervals are also reported. All costs have been adjusted for inflation using the Reserve Bank of Australia's inflation calculator,^29^ and are reported in constant prices (AUD 2023/24). For reference, 1AUD = 0.64USD; 0.49GBP; 0.58EUR (May 2025). Medication costs have been analyzed from the Commonwealth Government's perspective (i.e., the PBS funder's perspective).

An assessment of any inequality in access to antiemetic medications was conducted by plotting concentration curves and calculating concentration indices for each antiemetic medication (metoclopramide, ondansetron, and prochlorperazine). Concentration curves represent the cumulative proportion of a health variable on the y‐axis (for this study, we plot both the quantity of medication dispensed, and Government expenditure for each medication) as a function of the cumulative proportion of the population on the x‐axis, ranked according to socioeconomic status (measured using IRSD deciles) beginning with the most disadvantaged groups and ending with the least disadvantaged.^30^ These graphs visually represent the socioeconomic distribution for each antiemetic medication. A 45‐degree line through the origin is added to the graph to represent perfect equality. When the concentration curve lies above this line, the outcome of interest (i.e., medication access or Government expenditure) is disproportionately concentrated among more disadvantaged women; when it lies below the line, it is more concentrated among less disadvantaged women. The further the curve deviates from the 45‐degree line, the greater the inequality. A concentration index is then calculated for each curve, providing a numerical measure of the extent of inequality in each distribution. The index ranges from −1 to +1, with negative values indicating a pro‐poor distribution (i.e., a curve above the line of equality), and positive values indicating a pro‐rich distribution (i.e., a curve below the line of equality). The formula used to calculate the concentration index is the convenient regression formula:

$$2\sigma_{r}^{2}\left(\frac{h_{i}}{\mu }\right)=\alpha +\beta r_{i}+\varepsilon_{i}$$

In the above equation, $\sigma_{r}^{2}$ = variance of the fractional rank, *h*_*i*_ = the health sector variable (quantity of medications accessed, or public expenditure on medication), μ = mean of the health sector variable, *r*_*i*_ = the fractional rank of each individual (*i*) in the socioeconomic distribution, α = intercept, $\varepsilon_{i}$ = error term, and β = ordinary least squares regression estimate of the concentration index (C).^30^

To account for the lack of independence resulting from women contributing more than one pregnancy to the dataset during the study timeframe, standard errors were adjusted for clustering at the woman‐level using one‐way cluster‐robust (Huber‐White sandwich) variance estimation. Individual patient‐level outcome data, with area‐level IRSD ranks used as a proxy for socioeconomic disadvantage, were used to calculate the concentration index. Concentration curves have been plotted using IRSD deciles (grouped data). Where the IRSD is reported elsewhere in this study, it is presented in IRSD quintiles to aid readability of the results.

Concentration indices were evaluated based on effect sizes and 95% confidence intervals, with *p*‐values reported to indicate whether indices differed significantly from zero using a Bonferroni‐adjusted significance threshold of *p* < 0.0083 (two‐sided) to account for multiple statistical hypothesis tests (*n* = 6). Results were primarily interpreted based on effect size magnitude and policy relevance, rather than relying on *p*‐values as the sole indicators of importance.

## 3 RESULTS

### 3.1 Demographic characteristics

Demographic characteristics for pregnant women are reported in Table 2, stratified according to socioeconomic quintile. The proportion of missing data was small (<0.4% across all demographic variables, except BMI (<1.6%)).

**TABLE 2:** Demographic characteristics of pregnant women, Queensland, Australia, 2013/14 to 2017/18; stratified by socioeconomic quintile.

Maternal characteristics	Most disadvantage Q1	Q2	Q3	Q4	Least disadvantage Q5	Total
Total	54 579 (18.3%)	48 703 (16.4%)	75 815 (25.5%)	73 357 (24.6%)	45 176 (15.2%)	297 630 (100%)
*Maternal age*						
<20 years	2909 (5.3%)	1898 (3.9%)	1972 (2.6%)	1046 (1.4%)	335 (0.7%)	8160 (2.7%)
20–<35 years	42 907 (78.6%)	37 600 (77.2%)	58 396 (77.0%)	53 582 (73.0%)	29 569 (65.5%)	222 054 (74.6%)
>35 years	8763 (16.1%)	9205 (18.9%)	15 447 (20.4%)	18 729 (25.5%)	15 272 (33.8%)	67 416 (22.7%)
Missing	0 (0.0%)	0 (0.0%)	0 (0.0%)	0 (0.0%)	0 (0.0%)	0 (0.0%)
*Country of birth*						
Australia	11 874 (21.8%)	10 625 (21.8%)	19 866 (26.2%)	23 832 (32.5%)	14 251 (31.6%)	80 448 (27.0%)
Other	42 703 (78.2%)	38 074 (78.2%)	55 949 (73.8%)	49 514 (67.5%)	30 925 (68.5%)	217 165 (73.0%)
Missing	2 (0.0%)	4 (0.01%)	0 (0.0%)	11 (0.01%)	0 (0.0%)	17 (0.01%)
*Indigenous status*						
Yes	6521 (12.0%)	5086 (10.4%)	3734 (4.9%)	1524 (2.1%)	564 (1.3%)	17 429 (5.9%)
No	48 057 (88.1%)	43 614 (89.6%)	72 081 (95.1%)	71 829 (97.9%)	44 609 (98.7%)	280 190 (94.1%)
Missing	1 (<0.01%)	3 (0.01%)	0 (0.0%)	4 (0.01%)	3 (0.01%)	11 (<0.01%)
*Gravidity*						
Not first pregnancy	40 303 (73.8%)	34 448 (70.7%)	52 366 (69.1%)	50 100 (68.3%)	29 459 (65.2%)	206 676 (69.4%)
First pregnancy	14 274 (26.2%)	14 255 (29.3%)	23 449 (30.9%)	23 257 (31.7%)	15 717 (34.8%)	90 952 (30.6%)
Missing	2 (<0.01%)	0 (0.0%)	0 (0.0%)	0 (0.00%)	0 (0.0%)	2 (<0.01%)
*Plurality N (%)*						
Multiple	753 (1.4%)	683 (1.4%)	1132 (1.5%)	1096 (1.5%)	797 (1.8%)	4461 (1.5%)
Singleton	53 826 (98.6%)	48 020 (98.6%)	74 683 (98.5%)	72 261 (98.5%)	44 379 (98.2%)	293 169 (98.5%)
Missing	0 (0.0%)	0 (0.0%)	0 (0.0%)	0 (0.00%)	0 (0.0%)	0 (0.00%)
**Smoking status**						
*Before 20 weeks*						
Yes	11 472 (21.0%)	7831 (16.1%)	8331 (11.0%)	5063 (6.9%)	1835 (4.1%)	34 532 (11.6%)
No	42 896 (78.6%)	40 676 (83.5%)	67 327 (88.8%)	68 138 (92.9%)	43 260 (95.8%)	262 297 (88.1%)
Missing	211 (0.4%)	196 (0.4%)	157 (0.2%)	156 (0.2%)	81 (0.2%)	801 (0.3%)
*Medical conditions*						
Yes	17 179 (31.5%)	13 881 (28.5%)	21 274 (28.1%)	21 937 (29.9%)	15 758 (34.9%)	90 029 (30.3%)
No	37 395 (68.5%)	34 821 (71.5%)	54 539 (71.9%)	51 420 (70.1%)	29 418 (65.1%)	207 593 (69.7%)
Missing	5 (0.01%)	1 (<0.01%)	2 (<0.01%)	0 (0.00%)	0 (0.0%)	8 (<0.01%)
*Pregnancy complication*						
Yes	38 129 (69.9%)	33 750 (69.3%)	53 282 (70.3%)	51 640 (70.4%)	33 017 (73.1%)	209 818 (70.5%)
No	16 446 (30.1%)	14 952 (30.7%)	22 532 (29.7%)	21 716 (29.6%)	12 159 (26.9%)	87 805 (29.5%)
Missing	4 (0.01%)	1 (<0.01%)	1 (<0.01%)	1 (<0.01%)	0 (0.0%)	7 (<0.01%)
*BMI category*						
Underweight	3078 (5.6%)	2658 (5.5%)	3915 (5.2%)	4253 (5.8%)	2548 (5.6%)	16 452 (5.5%)
Healthy weight	22 606 (41.4%)	22 918 (47.1%)	37 213 (49.1%)	41 157 (56.1%)	28 112 (62.2%)	152 006 (51.1%)
Overweight	13 004 (23.8%)	11 553 (23.7%)	17 871 (23.6%)	15 898 (21.7%)	8853 (19.6%)	67 179 (22.6%)
Obese	15 019 (27.5%)	10 874 (22.3%)	15 633 (20.6%)	11 288 (15.4%)	5088 (11.3%)	57 902 (19.5%)
Missing	872 (1.6%)	700 (1.4%)	1183 (1.6%)	761 (1.0%)	575 (1.3%)	4091 (1.4%)
*Assisted conception*						
Yes	1569 (2.9%)	1915 (3.9%)	3600 (4.7%)	4694 (6.4%)	4062 (9.0%)	15 840 (5.3%)
No	53 008 (97.1%)	46 785 (96.1%)	72 214 (95.3%)	68 662 (93.6%)	41 114 (91.0%)	281 783 (94.7%)
Missing	2 (<0.01%)	3 (0.01%)	1 (<0.01%)	1 (<0.01%)	0 (0.0%)	7 (<0.01%)
*Charging status for hospital admission*						
Public	46 497 (85.2%)	37 093 (76.1%)	53 330 (70.3%)	47 668 (65.0%)	21 202 (46.9%)	205 790 (69.1%)
Private	7984 (14.6%)	11 534 (23.7%)	22 347 (29.5%)	25 498 (34.7%)	23 860 (52.8%)	91 223 (30.7%)
Missing	98 (0.2%)	76 (0.2%)	138 (0.2%)	191 (0.3%)	114 (0.3%)	617 (0.2%)

### 3.2 Quantity and prevalence of antiemetic medications dispensed to pregnant women via the Pharmaceutical Benefits Scheme (PBS)

Table 3 reveals that metoclopramide accounted for the majority (78.7%) of PBS‐listed antiemetics dispensed to pregnant women. Ondansetron was the second most frequently dispensed (19.2%), despite not being PBS‐approved for nausea and vomiting of pregnancy. Prochlorperazine represented just 2.1% of all dispensings. The highest volume of antiemetic dispensings occurred in the middle socioeconomic quintile (Q3), reflecting the higher number of pregnancies in this group. Notably, when prevalence was calculated to account for differences in the underlying number of pregnancies within a socioeconomic quintile, results for all three medications were highest among women in the most socioeconomically disadvantaged quintiles (Q1 to Q3) and declined progressively across the two least disadvantaged groups (Q4 and Q5). Over the study timeframe, prevalence of access to ondansetron and metoclopramide increased steadily, while prochlorperazine access remained consistently low (see Table S1 in Supporting Information).

**TABLE 3:** Quantity, prevalence, and government expenditure on antiemetic medications dispensed to pregnant women according to socioeconomic quintile, Queensland, Australia, 2013/14 to 2017/18, constant prices (2023/2024 Australian dollars).

IRSD Quintile	Number of pregnancies (column%)	Metoclopramide				Ondansetron				Prochlorperazine			
		Number of prescriptions dispensed (column%)	Prevalence (≥1 dispensing per pregnancy)	Total sum Government expenditure (column%)	Mean Government cost per dispensing (95% CI)	Number of prescriptions dispensed (column%)	Prevalence (≥1 dispensing per pregnancy)	Total sum Government expenditure (column%)	Mean Government cost per dispensing (95% CI)	Number of prescriptions dispensed (column%)	Prevalence (≥1 dispensing per pregnancy)	Total sum Government expenditure (column%)	Mean Government cost per dispensing (95% CI)
Q1 Most disadvantage	54 579 (18.3%)	16 968 (21.9%)	21.0% (11 462/54 579)	$49 570 (33.9%)	$2.92 (2.86–2.98)	4132 (21.9%)	3.8% (2058/54 579)	$51 344 (29.7%)	$12.43 (12.01–12.84)	458 (21.7%)	0.7% (364/54 579)	$1122 (27.3%)	$2.45 (2.12–2.78)
Q2	48 703 (16.4%)	13 484 (17.4%)	19.1% (9318/48 703)	$31 932 (21.9%)	$2.37 (2.31–2.43)	3475 (18.4%)	3.6%(1759/48 703)	$40 387 (23.4%)	$11.62 (11.08–12.16)	371 (17.5%)	0.6% (308/48 703)	$961 (23.4%)	$2.59 (2.17–3.01)
Q3	75 815 (25.5%)	20 379 (26.3%)	19.1% (14 481/75 815)	$36 004 (24.6%)	$1.77 (1.72–1.81)	5085 (27.0%)	3.8% (2912/75 815)	$42 456 (24.6%)	$8.35 (8.00–8.70)	596 (28.2%)	0.6% (459/75 815)	$1374 (33.4%)	$2.30 (1.93–2.68)
Q4	73 357 (24.7%)	17 507 (22.6%)	17.2% (12 612/73 357)	$21 933 (15.0%)	$1.25 (1.21–1.29)	4157 (22.0%)	3.4% (2466/73 357)	$25 766 (14.9%)	$6.20 (5.84–6.55)	430 (20.3%)	0.5% (345/73 357)	$450 (11.0%)	$1.05 (0.81–1.28)
Q5 Least disadvantage	45 176 (15.2%)	9059 (11.7%)	14.9% (6747/45 176)	$6731 (4.6%)	$0.74 (0.70–0.79)	2005 (10.6%)	2.7% (1233/45 176)	$13 013 (7.5%)	$6.49 (5.85–7.13)	260 (12.3%)	0.5% (210/45 176)	$201 (4.9%)	$0.77 (0.52–1.03)
Total	297 630 (100%)	77 397 (100%)	18.4% (54 620/297 360)	$146 171 (100%)	$1.89 (1.87–1.91)	18 854 (100%)	3.5% (10 428/297 630)	$172 966 (100%)	$9.17 (8.98–9.37)	2115 (100%)	0.6% (1686/297 630)	$4108 (100%)	$1.94 (1.78–2.10)
% Of total antiemetic dispensings		78.7% (77 397/98 366)				19.2% (18 854/98 366)				2.1% (2115/98 366)			
% Of total Government expenditure on antiemetic dispensings		45.2% ($146 171/$323 245)				53.5% ($172 966/$323 245)				1.3% ($4108/$323 245)			

Abbreviations: IRSD, index of relative socioeconomic disadvantage; 95%CI, 95% confidence interval.

### 3.3 Government expenditure on antiemetics dispensed during pregnancy

Table 3 shows government expenditure on antiemetics dispensed during pregnancy was highest for ondansetron, accounting for AUD $172 966 (53.5%) of total PBS expenditure, despite its lack of PBS approval for NVP, indicating substantial off‐label use and leakage of public funds. Metoclopramide accounted for a smaller share of Government expenditure (45.2%), while prochlorperazine represented only 1.3% of total PBS expenditure in this category. These expenditure patterns partly reflect differences in total medication costs (i.e., the combined patient contribution and public subsidy), with prochlorperazine being the least expensive, followed by metoclopramide, and ondansetron being the most costly. For prochlorperazine and metoclopramide, patients without a concession card often pay the full medication cost, resulting in substantially lower *average* government subsidies per dispensing compared with ondansetron. This pattern is consistent with the equity elements embedded within the PBS, whereby patients with a lower capacity to pay (i.e., concessional patients) incur lower out‐of‐pocket expenses and greater public subsidy. Table 3 shows the influence concessional status has on reducing mean government cost per dispensing, with the proportion of antiemetic dispensings to concessional patients known to increase as socioeconomic disadvantage increases (Q1: 50.9% vs. Q5: 14.9%). The influence of price reductions due to patients reaching the safety net was minimal in our patient population, occurring in only 0.3% of all antiemetic dispensings.

### 3.4 Socioeconomic dispersion of antiemetic medications dispensed to pregnant women

**FIGURE 1:**
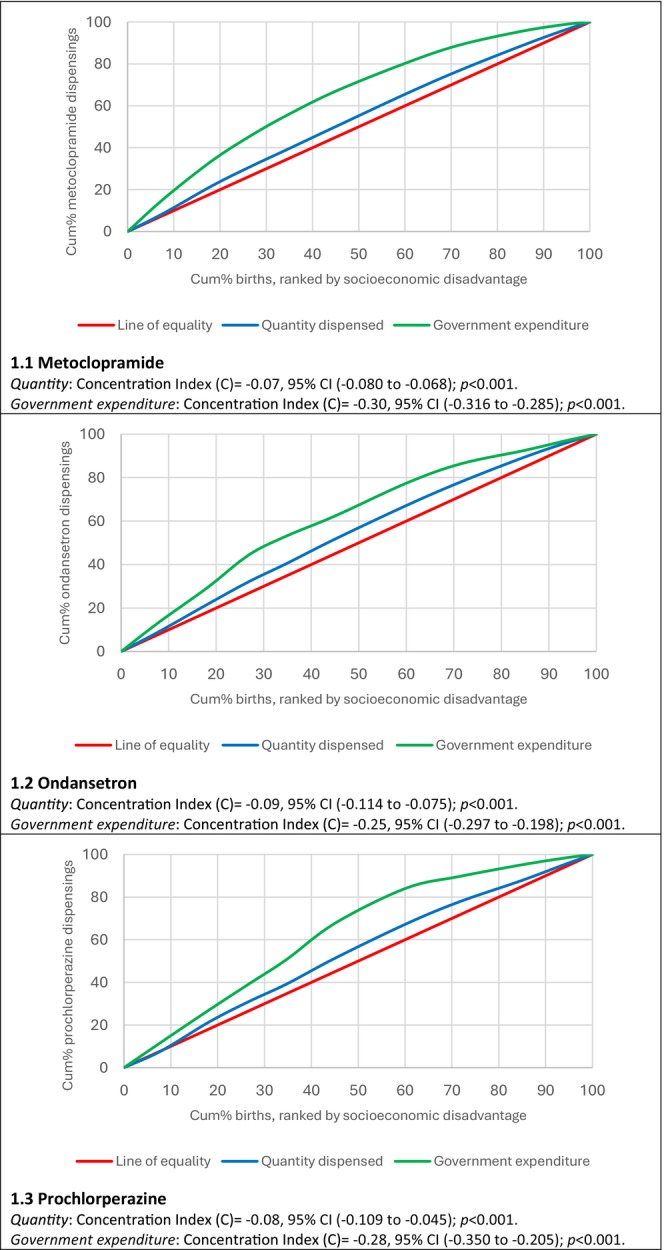
Concentration curve showing the socioeconomic dispersion of Pharmaceutical Benefits Scheme funded antiemetic medications dispensed to pregnant women, Queensland, Australia, 2013/14–2017/18.

Concentration curves for metoclopramide, ondansetron, and prochlorperazine indicated a small degree of pro‐poor inequality in access (all C < −0.10; Figure 1). In contrast, concentration curves for Government expenditure revealed a moderate degree of pro‐poor inequality in the distribution of public spending (Figure 1). The poorest 50% of pregnant women accounted for approximately 70% of total PBS expenditure on these medications, indicating a notable concentration of public expenditure among more socioeconomically disadvantaged groups. This pattern was reflected in concentration index estimates for Government expenditure that were moderate in magnitude (C_metoclopramide_ = −0.30, 95% CI (−0.316 to −0.285); C_ondansetron_ = −0.25, 95% CI (−0.297 to −0.198); C_prochlorperazine_ = −0.28, 95% CI (−0.350 to −0.205); *p* < 0.001 for each distribution).

## 4 DISCUSSION

This study found that ondansetron accounted for the highest public expenditure on antiemetics dispensed to pregnant women under Australia's PBS, despite not being approved by the Therapeutic Goods Administration or the PBS for managing NVP. This reflects the substantial barriers faced by prescribers and pregnant women in accessing essential treatment for even the most common pregnancy‐related conditions. It also reflects the workaround strategies, such as off‐label prescribing, that clinicians may adopt to navigate complex regulatory constraints. These practices often prioritize the patient‐prescriber relationship and demonstrate empathy for socioeconomic hardship, enabling pregnant women to access the care they need. They may also reflect efforts to manage severe symptoms in the community, potentially avoiding costly hospital admissions where ondansetron might otherwise be accessed.

Beyond the need for clinicians to navigate non‐standard access pathways for managing NVP, we found a pro‐poor distribution for all PBS‐dispensed antiemetics, with both access and public expenditure concentrated among women residing in lower‐income areas. A small degree of socioeconomic inequality was observed for quantity dispensed (concentration indices < −0.10; *p* < 0.001). Importantly, these findings must be interpreted in terms of effect size rather than statistical significance alone. In comparison, the distribution of public expenditure to support medication access showed a moderate degree of pro‐poor inequality (concentration indices > −0.25; *p* < 0.001). While this pattern aligns with the equity design elements embedded in the PBS, whereby concessional patients receive larger subsidies, it may also reflect differential use of non‐PBS‐listed first‐line treatments (eg ginger, pyridoxine, doxylamine). These therapies are subject to full out‐of‐pocket payments, posing greater financial barriers for lower‐income women compared to wealthier women who may be better positioned to access them privately. Wealthier women are also better positioned to access second‐line therapies like ondansetron via private prescribing. Additionally, private prescriptions for larger quantities of antiemetics may reduce the price per tablet for general patients, potentiating unequal access outside PBS provisions. We therefore argue that PBS‐supplied access to medications for NVP is skewed by regulatory misalignment with clinical guidelines, limiting equitable access to necessary medicines. Future analyses that incorporate an accurate indicator of clinical need would enable a benefit incidence analysis, helping to distinguish whether the observed distribution patterns reflect differences in underlying clinical need or are potentially driven by differential patterns of prescription‐seeking behavior.

More broadly, our findings highlight ongoing challenges in medication access during pregnancy, especially for NVP—a condition historically linked to the evolution of pharmaceutical regulation. Legal and regulatory complexities can delay treatment, compromise quality of life, and contribute to broader societal burdens including economic hardship for families. Prescribers are left to navigate a legal and ethical landscape that is fraught with liability concerns, often resorting to off‐label prescribing that lacks the standard regulatory safeguards. This places both prescribers and pregnant women in precarious positions, where treatment decisions are made without robust safety and efficacy data. Importantly, however, on‐label access requires a pharmaceutical company to actively apply for indication‐specific approval through standard Therapeutic Goods Administration regulatory pathways. When a medicine is off‐patent and already widely used off‐label in practice (e.g. ondansetron for NVP), the commercial incentive to pursue such approval is limited; especially for pregnancy‐related indications, since pregnant women represent a relatively small proportion of the population. It is somewhat ironic that the condition which prompted modern drug safety reforms remains neglected in terms of therapeutic innovation. While thalidomide's legacy rightly includes enhanced regulatory oversight, it has also inadvertently constrained pharmaceutical research and development in maternal health.^31^ Therapeutic options for NVP remain limited, safety data are insufficient, and access can be inequitable. These systemic challenges underscore the urgent need for policy reform. A more nuanced approach is essential; one that upholds rigorous safety standards while actively fostering pharmaceutical innovation and ensuring equitable access to woman‐centered maternal healthcare.

While there is limited evidence describing the socioeconomic dispersion of antiemetic medications to manage NVP, several international studies offer useful comparisons. In the United States, ondansetron prescribing for NVP more than doubled between 2008 and 2013, despite no approval from the Food and Drug Administration for this indication.^32^ In Switzerland, mandatory (private) health insurance claims system data (2014 to 2018) showed antinausea medications were dispensed in 16.4% of pregnancies, with metoclopramide most common (14.4%) and ondansetron used in only 1.3% of pregnancies.^33^ Off‐label prescribing of ondansetron by general practitioners in the United Kingdom has shown similar patterns, increasing from 0.1% of pregnancies in 2005 to 2.5% in 2019.^16^ Notably, this finding occurred in a health system that removes financial barriers to medication access during pregnancy,^34^ suggesting the observed patterns may also be reflective of broader prescribing trends and guidelines rather than medication affordability alone. In contrast, United States data from 2014 showed ondansetron was the most prescribed antiemetic (22.2%), compared to 3.2% for metoclopramide.^17^ Our study found metoclopramide was accessed by 18.4% of pregnant women via the PBS, with a mild but consistent increase over time. Ondansetron was accessed in 3.5% of pregnancies, and prochlorperazine in 0.6%. Notably, our data reflects PBS‐dispensed medications only, excluding private prescriptions and hospital inpatients. We identified 18 854 PBS‐dispensed ondansetron prescriptions, indicating substantial off‐label use and PBS‐leakage. An Australian study has previously described off‐label prescribing of PBS‐subsidized ondansetron during pregnancy, finding its use was more common among privately insured women (OR: 5.8).^19^ Together, these findings highlight evolving prescribing patterns during pregnancy and underscore the need for clarity in regulatory guidance and more equitable access to recommended pharmacotherapies.

This study was subject to several assumptions and limitations. Firstly, we assumed that all antiemetics dispensed during pregnancy were indeed accessed solely for the management of NVP. Secondly, our findings are subject to potential misclassification bias due to the ecological fallacy, as socioeconomic status was inferred from the woman's usual place of residence. This approach may not accurately reflect individual socioeconomic circumstances, and we also assumed that place of residence remained unchanged throughout pregnancy. Third, limitations in the dataset made it challenging to assign a precise pregnancy start date, as only the month and year of delivery was available. Antiemetics are most dispensed in the first trimester, minimizing the risk of missing prescriptions in the final month of pregnancy; however, some early pregnancy dispensings may have been missed. This risk is heightened by our exclusion of the first 30 days of pregnancy, and we therefore acknowledge that our analysis likely underestimates the prevalence and volume of PBS‐dispensed antiemetics during pregnancy. Fourth, since the QPDC dataset omits pregnancies that end before 20 weeks' gestation, we are unable to capture early antiemetic use for NVP among these pregnancies. This is an important limitation, given that the first trimester is both a particularly sensitive period for fetal development and the stage in which antiemetics are used most frequently. Fifth, our analysis includes prescriptions dispensed under the CTG scheme, which provides additional co‐payment reductions for Aboriginal and Torres Strait Islander patients with or at risk of chronic disease. As Indigenous status is more prevalent in disadvantaged groups, this likely increased the average public subsidy amount for antiemetics dispensed to these women. Additionally, the concentration of Indigenous Australians varies across states, and our dataset only includes births occurring in Queensland. Sixth, concessional versus general patient status was unavailable for all women, limiting more in‐depth analysis. We also acknowledge that for general patients, under co‐payment prescriptions may be cheaper via private prescription than via the PBS, contributing to a pro‐poor distribution. Seventh, the study design carries inherent limitations, including missing data, potential linkage errors, and variation in the consistency and accuracy of recorded information due to differences in personnel and coding practices. Eighth, our analyses have not accounted for the influence of different pack sizes for antiemetics. For example, metoclopramide and prochlorperazine are typically dispensed in a pack size of 25 tablets, while ondansetron is typically dispensed in a quantity of either 4 or 10 tablets. The quantity prescribed, however, is subject to various PBS prescribing nuances, which were considered outside the scope of this study. Ninth, we recognize that multiple statistical comparisons increase the risk of a Type I error (incorrectly concluding that socioeconomic inequality exists when it does not), and that large observational datasets can yield statistically significant results for findings of very small magnitude. Consequently, results were interpreted with consideration of the inequality magnitude and policy relevance rather than statistical significance alone. Finally, as these findings are situated within the Australian pharmaceutical policy environment, their applicability to health systems with substantially different regulatory frameworks or financing arrangements may be limited.

## 5 CONCLUSION

This study offers important insights into the complexities of accessing antiemetics to manage NVP in Australia. Despite not being approved for NVP through the PBS, ondansetron accounted for the highest public expenditure on antiemetics, highlighting the disconnect between regulatory and policy frameworks and clinical practice. We also found a pro‐poor distribution in both access to and public expenditure on PBS‐supplied metoclopramide, ondansetron, and prochlorperazine. While this aligns with the progressive nature of PBS financing, it also points to a regulatory environment that limits access to non‐subsidized (but recommended) therapies for socioeconomically disadvantaged groups. Furthermore, historical factors, including the thalidomide tragedy, have constrained therapeutic innovation in this space. These entrenched, systemic challenges underscore the urgent need for policy reform and sustained research investment to ensure *all* pregnant women can access necessary medications. Without such action, disparities in access will persist and opportunities for robust post‐marketing surveillance using linked administrative data will remain limited—ultimately impacting the health and well‐being of women and their families.

## AUTHOR CONTRIBUTIONS

HJ and EC conceptualized the study. HJ and EC led the data analysis, which was conducted by HJ under the guidance of EC, with additional methodological input from JE. HJ drafted the complete manuscript, which was reviewed and edited by all study authors (LG, JE, SW, EC) in an iterative manner, including input on statistical analysis, data presentation, and interpretation of the results. All authors reviewed and approved the final version of the manuscript and accept responsibility for the integrity of the research.

## FUNDING INFORMATION

H.J. was supported by an Australian Government Research Training Program Stipend (RTPS) from the University of Technology Sydney. E.C. was supported by a National Health and Medical Research Council (NHMRC) Fellowship (APP1159536). L.E.G. was supported by a Channel 7 Children's Research Foundation Fellowship (CRF‐210323). No funding bodies played any role in the study design, conduct, data collection, data analysis, interpretation, or preparation of this manuscript.

## CONFLICT OF INTEREST STATEMENT

E.C. reports being a member of the Australian Federal Government's Pharmaceutical Benefits Advisory Committee Economics Subcommittee. No other authors have any conflicts of interest to disclose.

## ETHICS STATEMENT

Ethics approval was obtained from the Townsville Hospital and Health Service Human Research Ethics Committee (HREC) (HREC/16/QTHS/223; approved November 2016), the Australian Institute of Health and Welfare HREC (EO2017‐1‐338; approved May 2017), and the University of Technology Sydney (UTS HREC REF NO. ETH23‐8862; approved December 2023). Public Health Act approval (RD007377; approved April 2017) was also obtained for the study.

## Supporting information


**Table S1:** Prevalence (≥1 dispensing per pregnancy) of Pharmaceutical Benefits Scheme listed antiemetic medications dispensed to pregnant women according to socioeconomic quintile, Queensland, Australia, 2013/14 to 2017/18.

## Data Availability

The datasets analyzed for this study are not publicly available due to the strict ethics and privacy criteria that govern access to the data repository but are available from the senior author (EC) on appropriate request.

## References

[1] Louik C , Hernandez‐Diaz S , Werler MM , Mitchell AA . Nausea and vomiting in pregnancy: maternal characteristics and risk factors. Paediatr Perinat Epidemiol. 2006;20(4):270‐278. 16879499 10.1111/j.1365-3016.2006.00723.x

[2] Lacroix R , Eason E , Melzack R . Nausea and vomiting during pregnancy: a prospective study of its frequency, intensity, and patterns of change. Am J Obstet Gynecol. 2000;182(4):931‐937. 10764476 10.1016/s0002-9378(00)70349-8

[3] Einarson TR , Piwko C , Koren G . Quantifying the global rates of nausea and vomiting of pregnancy: a meta analysis. J Popul Ther Clin Pharmacol. 2013;20(2):e171‐e183. 23863575

[4] Bottone‐Post C . Chapter 11: nausea and vomiting of pregnancy. In: Mattison D , Halbert L‐A , eds. Clinical Pharmacology During Pregnancy. 2nd ed. Academic Press; 2022:155‐176.

[5] Piwko C , Koren G , Babashov V , Vicente C , Einarson TR . Economic burden of nausea and vomiting of pregnancy in the USA. J Popul Ther Clin Pharmacol. 2013;20(2):e149‐e160. 23913638

[6] Birmingham Health Partners . Healthy Mum, Healthy Baby, Healthy Future: the Case for UK Leadership in the Development of Safe, Effective and Accessible Medicines for Use in Pregnancy. University of Birmingham; 2022.

[7] Heitmann K , Holst L , Lupattelli A , Maltepe C , Nordeng H . Treatment of nausea in pregnancy: a cross‐sectional multinational web‐based study of pregnant women and new mothers. BMC Pregnancy Childbirth. 2015;15(1):321. 26628289 10.1186/s12884-015-0746-2 PMC4667480

[8] Curran WJ . The thalidomide tragedy in Germany: the end of a historic medicolegal trial. N Engl J Med. 1971;284(9):481‐482. 5100423 10.1056/NEJM197103042840906

[9] Fisk NM , Atun R . Market failure and the poverty of new drugs in maternal health. PLoS Med. 2008;5(1):e22. 18215109 10.1371/journal.pmed.0050022 PMC2211556

[10] Alexe A , Wurst K , Balramsingh‐Harry L , et al. Points to consider on the use of medicines in pregnancy throughout the product lifecycle based on global regulatory guidance. Ther Innov Regul Sci. 2025;59(3):462‐470. 39987266 10.1007/s43441-024-00736-0 PMC12018506

[11] Tan A , Foran R , Henry A . Managing nausea and vomiting in pregnancy in a primary care setting. Aust J Gen Pract. 2016;45(8):564‐568. 27610445

[12] Jackson H , Grzeskowiak L , Enticott J , Wise S , Callander E . How the structural determinants of health inequities impact access to prescription medication for pregnant women in Australia: a narrative review. Lancet Reg Health West Pac. 2024;42:100934. 38357390 10.1016/j.lanwpc.2023.100934 PMC10865029

[13] Little MO , Wickremsinhe MN . Research with pregnant women: a call to action. Reprod Health. 2017;14(3):156. 29297373 10.1186/s12978-017-0419-x PMC5751672

[14] Koblinsky M , Moyer CA , Calvert C , et al. Quality maternity care for every woman, everywhere: a call to action. Lancet. 2016;388(10057):2307‐2320. 27642018 10.1016/S0140-6736(16)31333-2

[15] Dey T , Widmer M , Coomarasamy A , et al. Advancing maternal and perinatal health through clinical trials: key insights from a WHO global consultation. Lancet Glob Health. 2025;13(4):e740‐e748. 40155111 10.1016/S2214-109X(24)00512-6

[16] Slattery J , Quinten C , Candore G , et al. Ondansetron use in nausea and vomiting during pregnancy: a descriptive analysis of prescription patterns and patient characteristics in UK general practice. Br J Clin Pharmacol. 2022;88(10):4526‐4539. 35483963 10.1111/bcp.15370 PMC9545331

[17] Taylor LG , Bird ST , Sahin L , et al. Antiemetic use among pregnant women in the United States: the escalating use of ondansetron. Pharmacoepidemiol Drug Saf. 2017;26(5):592‐596. 28220993 10.1002/pds.4185

[18] Zur RL . Protected from harm, harmed by protection: ethical consequences of the exclusion of pregnant participants from clinical trials. Res Ethics. 2023;19(4):536‐545.

[19] Colvin L , Gill AW , Slack‐Smith L , Stanley FJ , Bower C . Off‐label use of ondansetron in pregnancy in Western Australia. Biomed Res Int. 2013;2013:909860. 24396830 10.1155/2013/909860 PMC3874333

[20] Callander EJ , Fox H . What are the costs associated with child and maternal healthcare within Australia? A study protocol for the use of data linkage to identify health service use, and health system and patient costs. BMJ Open. 2018;8(2):e017816. 10.1136/bmjopen-2017-017816 PMC5829863 29437751

[21] Statistical Collections and Integration Unit . In: Queensland Health , ed. Queensland Perinatal Data Collection Manual for the Completion of Perinatal Data 2017–2018. Queensland Health; 2017.

[22] Department of Health and Aged Care . National Medicines Policy 2022. Commonwealth of Australia; 2022.

[23] Department of Health and Aged Care . About the PBS. 2025 January 14. Accessed February 10, 2025. https://www.pbs.gov.au/info/about‐the‐pbs#What_is_the_PBS

[24] Department of Health and Aged Care . PBS Expenditure and Prescriptions Report 1 July 2023 to 30 June 2024. 2024 December 23. Accessed February 10, 2025. https://www.pbs.gov.au/info/statistics/expenditure‐prescriptions/expenditure‐prescriptions‐report‐1‐july‐2023‐30‐june‐2024

[25] Lowe SA , Armstrong G , Beech A , et al. SOMANZ position paper on the management of nausea and vomiting in pregnancy and hyperemesis gravidarum. Aust N Z J Obstet Gynaecol. 2020;60(1):34‐43. 31657004 10.1111/ajo.13084

[26] ETG Complete: Gastrointestinal. Therapeutic Guidelines Ltd.; 2002 (Aug 2022 version).

[27] Australian Bureau of Statistics . Technical Paper: Socio‐Economic Indexes for Areas (SEIFA) 2016 (2033.0.55.001). ABS; 2016.

[28] Sax Institute . Conducting research in SURE. 2025. Accessed February 18, 2025. https://www.saxinstitute.org.au/solutions/sure/conducting‐research‐in‐sure

[29] Reserve Bank of Australia . Inflation Calculator. 2025. Accessed February 18, 2025. https://www.rba.gov.au/calculator/

[30] O'Donnell O , Doorslaer E , Wagstaff A , Lindelow M . Analyzing Health Equity Using Household Survey Data: A Guide to Techniques and Their Implementation. The World Bank; 2008.

[31] Waggoner MR , Lyerly AD . Clinical trials in pregnancy and the “shadows of thalidomide”: revisiting the legacy of Frances Kelsey. Contemp Clin Trials. 2022;119:106806. 35654303 10.1016/j.cct.2022.106806 PMC9420797

[32] Koren G . Treating morning sickness in the United States—changes in prescribing are needed. Am J Obstet Gynecol. 2014;211(6):602‐606. 25151184 10.1016/j.ajog.2014.08.017

[33] Gerbier E , Graber SM , Rauch M , et al. Use of drugs to treat symptoms and acute conditions during pregnancy in outpatient care in Switzerland between 2014 and 2018: analysis of Swiss healthcare claims data. Swiss Med Wkly. 2021;151:w30048. 34843179 10.4414/smw.2021.w30048

[34] Maternity Action . Free NHS Prescriptions and NHS Dental Care for Pregnant Women and New Mothers. Maternity Action; 2025. Accessed March 25, 2026. https://maternityaction.org.uk/advice/free‐nhs‐prescriptions‐and‐nhs‐dental‐care‐for‐pregnant‐women‐and‐new‐mothers/

