# Supplementary material for: Pharmaceutical policy and off‐label prescribing in pregnancy: A population‐based historical cohort study analyzing inequality in access to antiemetics within Australia's Pharmaceutical Benefits Scheme
Source: Acta Obstet Gynecol Scand. 2026 Jun 11:10.1111/aogs.70283. Online ahead of print. doi: 10.1111/aogs.70283 (PMC13394017; doi:10.1111/aogs.70283)
Supplement: Supplementary file 1 — Table S1: Prevalence (≥1 dispensing per pregnancy) of Pharmaceutical Benefits Scheme listed antiemetic medications dispensed to pregnant women according to socioeconomic quintile, Queensland, Australia, 2013/14 to 2017/18. [file AOGS-9999-0-s001.docx]

**SUPPORTING INFORMATION**

**Pharmaceutical policy and off-label prescribing in pregnancy: A population-based historical cohort study analysing inequality in access to antiemetics within Australia’s Pharmaceutical Benefit’s Scheme.**

**Table S1: Prevalence (≥1 dispensing per pregnancy) of Pharmaceutical Benefits Scheme listed antiemetic medications dispensed to pregnant women according to socioeconomic quintile, Queensland, Australia, 2013/14 to 2017/18.**

| **IRSD Quintile** | **2013/14** | **2014/15** | **2015/16** | **2016/17** | **2017/18** | **Total** |
| --- | --- | --- | --- | --- | --- | --- |
| **1) Metoclopramide** | | | | | | |
| 1 | 20.1%  (2327/11 552) | 19.9%  (2266/11 386) | 21.1%  (2300/10 914) | 21.6%  (2263/10 464) | 22.5%  (2306/10 263) | 21.0%  (11 462/54 579) |
| 2 | 17.8%  (1842/10 327) | 18.6%  (1900/10 225) | 19.3%  (1869/9685) | 20.2%  (1875/9293) | 20.0%  (1832/9173) | 19.1%  (9318/48 703) |
| 3 | 18.4%  (2838/15 463) | 17.8%  (2738/15 386) | 19.4%  (3013/15 507) | 20.1%  (2971/14 753) | 19.9%  (2921/14 706) | 19.1%  (14 481/75 815) |
| 4 | 15.9%  (2352/14 774) | 16.9%  (2409/14 291) | 17.3%  (2571/14 905) | 18.2%  (2677/14 753) | 17.8%  (2603/14 634) | 17.2%  (12 612/73 357) |
| 5 | 14.1%  (1291/9170) | 14.3%  (1293/9020) | 14.9%  (1382/9271) | 15.5%  (1406/9048) | 15.9%  (1375/8667) | 14.9%  (6747/45 176) |
| Total | 17.4%  (10 650/61 286) | 17.6%  (10 606/60 308) | 18.5%  (11 135/60 282) | 19.2%  (11 192/58 311) | 19.2%  (11 037/57 443) | 18.4%  (54 620/297 630) |
| **2) Ondansetron** | | | | | | |
| 1 | 2.3%  (265/11 552) | 2.7%  (305/11 386) | 3.8%  (415/10 914) | 4.8%  (506/10 464) | 5.5%  (567/10 263) | 3.8%  (2058/54 579) |
| 2 | 2.2%  (228/10 327) | 2.9%  (296/10 225) | 3.9%  (377/9685) | 4.5%  (416/9293) | 4.8%  (442/9173) | 3.6%  (1759/48 703) |
| 3 | 2.6%  (402/15 463) | 3.1%  (480/15 386) | 3.9%  (602/15 507) | 4.6%  (680/14 753) | 5.1%  (748/14 706) | 3.8%  (2912/75 815) |
| 4 | 2.3%  (346/14 774) | 3.0%  (426/14 291) | 3.5%  (515/14 905) | 3.8%  (557/14 753) | 4.3%  (622/14 634) | 3.4%  (2466/73 357) |
| 5 | 2.1%  (189/9170) | 2.7%  (243/9020) | 2.8%  (260/9271) | 3.2%  (292/9048) | 2.9%  (249/8667) | 2.7%  (1233/45 176) |
| Total | 2.3%  (1430/61 286) | 2.9%  (1750/60 308) | 3.6%  (2169/60 282) | 4.2%  (2451/58 311) | 4.6%  (2628/57 443) | 3.5%  (10 428/297 630) |
| **3) Prochlorperazine** | | | | | | |
| 1 | 0.8%  (89/11 552) | 0.8%  (88/11 386) | 0.7%  (72/10 914) | 0.6%  (60/10 464) | 0.5%  (55/10 263) | 0.7%  (364/54 579) |
| 2 | 0.7%  (72/10 327) | 0.5%  (52/10 225) | 0.8%  (77/9685) | 0.6%  (52/9293) | 0.6%  (55/9173) | 0.6%  (308/48 703) |
| 3 | 0.7%  (103/15 463) | 0.6%  (89/15 386) | 0.7%  (109/15 507) | 0.6%  (87/14 753) | 0.5%  (71/14 706) | 0.6%  (459/75 815) |
| 4 | 0.5%  (80/14 774) | 0.5%  (68/14 291) | 0.5%  (75/14 905) | 0.4%  (64/14 753) | 0.4%  (58/14 634) | 0.5%  (345/73 357) |
| 5 | 0.5%  (44/9170) | 0.4%  (34/9020) | 0.5%  (44/9271) | 0.5%  (41/9048) | 0.5%  (47/8667) | 0.5%  (210/45 176) |
| Total | 0.6%  (388/61 286) | 0.5%  (331/60 308) | 0.6%  (377/60 282) | 0.5%  (304/58 311) | 0.5%  (286/57 443) | 0.6%  (1686/297 630) |
| IRSD: Index of Relative Socioeconomic Disadvantage | | | | | | |
